# Increasing the Quality of Trial Reporting

**DOI:** 10.5812/nms.11875

**Published:** 2013-09-15

**Authors:** Hamid Salehiniya, Samane Sabet Birjandi

**Affiliations:** 1 Atherosclerosis and Coronary Artery Research Centre. Birjand University of Medical Sciences, Birjand, IR Iran; 2 Midwifery Department, Islamic Azad University Branch of Birjand, Birjand, IR Iran

**Keywords:** Quality, Clinical Trial, Reporting

Dear editor,

Clinical trial is defined as a prospective study to consider the effect and value of an intervention or a control in human (1). Improving the quality of a clinical trial study needs appropriate analytic methods (2).

One important aspect for clinical study is comparing two groups at the commencement of the study. In an experimental study, it is very important for the two groups under the study to be the same and have no difference at the beginning of the study. Because if there is any difference between the two groups at the baseline, the result can be attribute to the difference in baseline rather than the effect of the drug or intervention. So, it is necessary to insure that the two groups were similar at the baseline (2). For this purpose, presenting tables with demographic and other factors at the beginning of the study and comparing the two groups with report P value is essential (2). Random allocation does not guarantee that the two groups are the same at the baseline (1). So to improve the quality of the report, presenting descriptive results of the groups and comparing the groups with report P value would be useful (2, 3).

In the study titled “Effects of a Three-Stage Intervention Program on the Holistic Health Status of Patients with Drug Addiction after Discharge”, published in one of the previous issues of Nursing and Midwifery Studies Journal (4), the two groups under the study are not comparable at the baseline.

According to what was mentioned above, the result of the study can be influenced by the differences between two groups at the baseline and different prognosis.

In conclusion, for improving the result of the intervention, descriptive results and the similarity between two groups at the baseline must be reported.
